# Individual differences in impulsivity and need for cognition as potential risk or resilience factors of diabetes self-management and glycemic control

**DOI:** 10.1371/journal.pone.0227995

**Published:** 2020-01-29

**Authors:** Alexander Hadj-Abo, Sören Enge, Jörn Rose, Hagen Kunte, Monika Fleischhauer

**Affiliations:** 1 Department of Psychology, Faculty of Natural Sciences, Medical School Berlin, Berlin, Germany; 2 Diabetes Centrum Berlin, Berlin, Germany; Mahidol University, THAILAND

## Abstract

**Objective:**

Impulsivity is marked by insufficient reflection and forethought, whereas Need for Cognition (NFC) also referred to as cognitive motivation or intellectual engagement is marked by elaborated thinking. The aim of this study was to investigate the potential role of these personality traits as resilience or risk factors, respectively, in diabetes self-management and glycaemic control. Further, it was examined whether diabetes-specific self-efficacy could serve as a mediator of these relationships.

**Design/Measures:**

Data of 77 participants with type 2 diabetes was ascertained, using self-report instruments for NFC, impulsivity, diabetes-specific self-efficacy, and diabetes self-management. Glycemic control was assessed by the biomarker HbA_1c_.

**Results:**

While NFC was strongly positively associated with diabetes self-management and glycemic control, impulsivity showed a reverse pattern. Results of simple and serial mediation models showed that the effects on diabetes self-management and HbA1c of both, impulsivity and NFC, were mediated by self-efficacy.

**Conclusion:**

The moderate to high standardized coefficients suggests that NFC might be an important protective factor and impulsivity a possible risk factor for effective diabetes self-management and glycemic control. These traits could be applied for an easy-to-use questionnaire-based patient screening, enabling trait-tailored treatments and programs which in turn may lower economic and health costs associated with poor diabetes-care.

## Introduction

### Diabetes and secondary diseases

Diabetes mellitus is a chronic, metabolic disease which results from insulin resistance or defective insulin secretion [1]. It is characterized by hyperglycemia, with prolonged hyperglycemia strongly increasing the risk for a variety of diabetes-related diseases. Today, around 460 million individuals are living with diabetes mellitus and in 2030, it is expected that more than 570 million people will be affected [2].

Especially type 2 diabetes mellitus where 90% of diabetes patients belong to [3, 4], and that is marked by an inefficient insulin function (i.e., insulin resistance), is an ever-growing global health problem with prevalence and incidence rates growing yearly relatively independent of wealth or ethnicity [5]. Projected numbers of people with type 2 diabetes in Germany suggest an increase up to 12.3 million cases in 2040 [6].

Type 2 diabetes differs from type 1 diabetes etiologically, in terms of the prevalence of secondary diseases [7–9], and regarding the treatment approaches [10]. In the present study, we focus on type 2 diabetes.

Diabetes mismanagement, resulting in high blood glucose, can cause a host of secondary diseases. Risk for heart attacks in diabetes patients is elevated up to 200% [11]. Foot infections with, in severe cases, limb amputations as a result of insufficient glycemic control are not rare [12]. Diabetes is also one of the main reasons for kidney failure and strokes [13]. Further, diabetes is regarded as the highest risk factor for deadly strokes [14]. In 2015, 3.8 million deaths could be attributed to diabetes in combination with high blood sugar [12]. In addition to somatic secondary diseases there are also psychological disorders that are bi-directionally related to diabetes [15–18]. The most common are depression and impulse control disorders like intermittent explosive disorder and bulimia nervosa, with binge eating disorder being the most prevalent impulse control disorder [19–22]. Moreover, the economic consequences of diabetes mismanagement such as on statutory health insurance systems are serious [23]. Due to the individual and societal consequences of type 2 diabetes, it is important to identify individual factors, such as personality traits, that may enable a targeted treatment or training plan for patients potentially preventing costly secondary diseases through an improvement in self-management and, in turn, glycemic control. In the following diabetes self-management will be defined and personality factors that might improve or hinder diabetes self-management will be introduced.

### Diabetes self-management and related personality factors

Disease self-management is defined as “the individual´s ability to manage the symptoms, treatment, physical and psychosocial consequences and life style changes inherent in living with a chronic condition” [24]. Diabetes self-management encompasses treatment behaviors with which patients are regularly confronted like diet, physical activity, medication adherence and blood sugar control [25]. Since diabetes self-management was identified as a facilitator for better quality of life, glycemic control and diet [15, 26, 27], it is important to identify the role of personality traits which might exert a specific influence on diabetes self-management and glycemic control.

Previous research on the relationship between personality traits and diabetes disease focused mainly on the Big Five of the Five-Factor-Model of personality [28]. This line of research attempts to identify which traits are important for the emergence of type 2 diabetes and beneficial when dealing with diabetes care. However, mixed results have been observed across different studies [29–31]. Because the global nature of the Big Five factors seems to be suboptimal in identifying personality-specific variance in diabetes self-management [29], the present study specifically addresses the personality traits impulsivity and need for cognition (NFC), also referred to as typical intellectual engagement or cognitive motivation, as potential resilience or risk factors of diabetes self-management and glycemic control.

Impulsivity is marked by behavior lacking sufficient reflection and foresight. It has been associated with worse diabetes self-management in type 1 diabetics [32, 33]. For type 2 diabetes, an association between diabetes diagnosis and impulse control disorders (particularly eating disorders) was found [22] and impulsivity or related personality traits have been identified as potential risk factors for the development of type 2 diabetes and behaviors that are themselves risk factors for the later development of diabetes [29, 34–37]. There is also evidence that hypoglycemia can lead to increased impulsive behaviors [38–39]. Moreover, Eckstrand and colleagues [10] were able to attribute subjects’ increased motor impulsivity to food stimuli to higher insulin resistance [10]. Furthermore, lower insulin sensitivity was linked to higher delayed reward discounting that is associated with impulsivity [38], and in older type 2 patients deficits in inhibitory control, but not in other aspects of executive functioning or cognitive control, were observed [39, 40]. These prior findings lead to our assumption that trait impulsivity could impair diabetes self-management through a lack of planning and insufficient regulation of behavior.

A further promising personality trait in terms of diabetes self-management and glycemic control is NFC, referring to individual differences in intrinsic cognitive motivation or intellectual engagement. Individuals with high NFC show a higher engagement and pleasure in thinking [41], are more focused on task-relevant information, tend to be goal-oriented and persistent, and actively search for information in order to come to valid decisions or judgments [40, 41]. Furthermore, NFC is positively associated with conscientious behavior [42], exhibiting beneficial effects on diabetes care [29, 30, 43]. NFC-related persistence and goal-orientated behavior could aid diabetes self-management and effective diabetes care, as for example blood sugar needs to be monitored constantly. Furthermore, being focused on task-relevant information could foster adherence to medical instructions in the context of diabetes care. It has been demonstrated that type 2 diabetics with higher NFC were more likely to remember relevant written diabetes information than patients low in NFC [44]. Of note, NFC has been already identified as a protective health factor such as in terms of burnout [45].

A potential factor through which these traits might exert their influence over glycemic control and self-management is diabetes-specific self-efficacy. Self-efficacy popularized by Bandura [46] describes the confidence to overcome new or difficult challenges with one’s own abilities. Since patients are regularly faced with managing their illness in new and demanding or difficult situations, the belief to handle such situations is important for effective behavior implementation. Indeed, it has been repeatedly shown that self-efficacy facilitates glycemic control [47, 48] and diabetes self-management [47, 49, 50], while also acting as a mediator for depression, distress, physical activity and quality of healthcare [47, 51, 52]. These facilitating effects also seem to be independent of ethnicity [47, 53]. Moreover, self-efficacy has already been found to be a mediator for trait impulsivity regarding health risks like marihuana and alcohol abuse [54, 55]. This research suggests that a potential relationship between the personality trait impulsivity and diabetes self-management could be mediated by diabetes-specific self-efficacy. Moreover, self-efficacy may mediate the potential relationship between NFC and self-management, as a deeper elaboration of diabetes-relevant medical information, the rational decision-making style, and the goal-oriented behavior of individuals high in NFC [44] might foster their confidence in handling the disease (diabetes-specific self-efficacy), which in turn should have positive consequences for their diabetes self-management and glycemic control.

### Research questions and hypotheses

The purpose of this paper was to examine the relationships and pathways of NFC and impulsivity, respectively, with diabetes-specific self-efficacy and diabetes self-management (indicated by self-report and HbA_1c_, a biological indicator of glycemic control). Specifically, we assume that a higher level of impulsivity is associated with lower diabetes self-management (hypothesis 1) and expect that a higher level of NFC is associated with better diabetes self-management (hypothesis 2). We further hypothesize that diabetes-specific self-efficacy mediates the relationships tested in hypotheses 1 and 2 (hypothesis 3).

## Methods

### Participants and procedure

Data of 77 patients with type 2 diabetes was available for analysis (63.6% male, age mean ± *SD* 62.3 ± 11.9 years, range 35–83 years). Subjects were recruited in a time span of three months from the 23.07.2018 to the 21.10.2018 and were only included if they had been suffering from type 2 diabetes for at least one year. The link to the online survey was distributed via flyers which were allocated to patients of a diabetes medical center by the leading doctor. We further recruited people through an online forum using personalized messages. Forty-six participants (59.7%) were recruited from their medical practitioner, 24 (31.2%) online and 7 (9.1%) found out about the study via a friend or colleague. The online survey started with a detailed description of the goals and procedure of the survey. After participants had given written informed consent, self-report measures of impulsivity, NFC, self-efficacy, and diabetes self-management were assessed and a short scale of the Big-Five traits [56] was given. Furthermore, sociodemographic and illness-related information were assessed (i.e., age, sex, nationality, education, duration of disease, method of measuring blood glucose and method of medication). Participants did not receive any kind of compensation. The study was approved by the ethic committee of the Medical School Hamburg.

Blood sugar control in this sample was mostly done by test strips (n = 64, 83.1%), while only a few participants used urine test strips (n = 5, 6.5%) or the relatively new continuous glucose monitoring systems (n = 6, 7.8%). Two participants did not check their blood sugar at all (2.6%). The time that had passed since first diagnosis was on average 12.4 years (SD = 8.2). Glycemic control was indicated by HbA_1c_ and available for 72 patients with values ranging from 5 to 8% (M = 7.1%, *SD* = .65). Of the patients with information on the highest school leaving certificate (n = 75), 32 (42.7%) stated that they had completed high school or university, 36 (48%) stated that they had completed secondary school or vocational training, and 7 (9.3%) stated that they had completed primary school or had no school leaving certificate.

### Variables

#### BIS-11

We measured impulsivity with the German version of the Barratt-Impulsiveness-Scale (BIS-11) [32]. Barratt and colleagues included information from medical, psychological, behavioral and social perspectives on impulsivity to enable a holistic and dimensional approach to the concept of impulsivity. The scale comprises 30 items answered on a 4-point Likert-scale ranging from 1 (rarely/never) to 4 (almost always). A sum score was then calculated where higher values represent a higher level of impulsivity (maximum value = 120) [32].

#### NFC-K

To examine NFC, we used the need for cognition short scale (NFC-K) developed by Beißert, Köhler, Rempel, and Beierlein [57]. The scale consists of four items recorded on a seven-point scale ranging from 1 (completely inappropriate) to 7 (absolutely appropriate). Thus, the NFC sum score ranges from 4 to 28, with more positive scores indicating a stronger tendency to engage in and enjoy cognitive activity.

#### SDSCA

Diabetes self-management was measured with the German version of the Summary of Diabetes Self-Care Activities (SDSCA-G) [58]. The German version comprises 9 items that capture the frequency of various diabetes self-care behaviors regarding diet (3 items), physical activity, blood sugar control and foot care (each 2 items). On an eight-point Likert scale (0–7) the participants are asked to state on how many of the last seven days these self-care behaviors were performed. Beside the sum score, scores for the four sub-scales were calculated.

#### DMSES

Diabetes-specific self-efficacy was measured using the diabetes-management self-efficacy scale (DMSES) [59]. On a 10-step numeric scale, the scale captures the participants`confidence in their performance in 15 behaviors related to diabetes management. At the point in time of data acquisition there was no psychometrically evaluated German version of the DMSES, which is why the British version of the scale was translated into German. One native speaker and one student of English studies translated the scale to German and another native speaker executed the back translation. Furthermore, a pretest with three interviewed diabetes patients in terms of item clarity/ambiguity was conducted. Because the items are simply worded and the fact that the scale exhibited excellent internal consistency in the present sample (Cronbach´s α = .94) we assume that the scale reliably depicts diabetes-specific self-efficacy.

#### HbA_1c_

HbA_1c_ (in %) was used as measure of glycosylated hemoglobin representing the glycemic control of patients [60]. Individuals were clearly instructed to report their last HbA_1c_ that all three month is handed out with the hemogram parameters by the doctor and that should also be documented in their diabetes diary.

### Statistical analysis

All statistical analyses were calculated using IBM SPSS Statistics (Version 25). Considering the lack of outliers and skew and kurtosis not exceeding values of +/-1, normal distribution can be assumed for all considered variables [61]. We also used bootstrapped confidence intervals reducing potential risks from violation of normal distribution [61]. T-test and correlation analyses were used to test for potentially confounding effects of socio-demographic or treatment variables. Next, we used ANOVAs and correlation analysis (Pearson’s *r)* to test hypotheses 1 and 2. To test hypothesis 3, mediation models were run using the PROCESS Plugin [62] for SPSS. In Model 1 impulsivity and in Model 2 NFC was used as independent variable. In both models, diabetes self-management was specified as dependent variable and diabetes-specific self-efficacy as mediator. Based on these models we further ran two serial mediation models in which diabetes-specific self-efficacy represented the first mediator and diabetes self-management the second, while glycemic control (HbA_1c_) represented the dependent variable. Our sample size, together with an alpha of .05 and a power of .80, enabled us to detect effects of medium size in the mediated regression analyses [63].

## Results

### Descriptives and control variables

Table 1 depicts the characteristics of the self-report scales. Internal consistencies were excellent for the BIS-11 sum score measuring general impulsivity (Cronbach’s *α* = .95) and for the DMSES measuring diabetes specific self-efficacy (*α* = .94). Acceptable to good internal consistency was obtained for the SDSCA main score (*α* = .74), measuring diabetes self-management. For NFC, measuring intellectual engagement or cognitive motivation, respectively, a good to excellent internal consistency of Cronbach’s *α* = .88 was obtained.

**Table 1:** Means, standard deviations and internal consistency’s of the self-report instruments.

Instruments (Items)	*M*	*SD*	Cronbach’s α
BIS-11 (30) ^a^	65.14	17.65	.95
DMSES (15) ^b^	117.17	34.75	.94
SDSCA (9) ^c^	39.64	11.37	.74
NFC-K (4)^d^	15.04	6.17	.88
HbA_1c_ (%) (1)	7.08	.65	-

*Annotations*.

^a^ Min = 1, Max = 4;

^b^ Min = 1, Max = 10;

^c^ Min = 0, Max = 7;

^d^ Min = 1, Max = 7.

BIS-11 = Barrat impulsiveness scale, SDSCA = Summary of Diabetes Self-care Activities Measure, DMSES = diabetes management self-efficacy scale, NFC-K = Need for cognition short scale

To test for gender-specific differences in NFC, impulsivity and self-efficacy, t-tests for independent variables were run. We found significant differences in NFC [*t* (75) = −3.03, *p* = .003] and impulsivity [*t* (75) = 3.33, *p* = .001], while no significant differences were found for self-efficacy [*t* (75) = −1.74, *p* = .089] and self-management [*t* (75) = − 1.25, *p* = .215]. Additionally, correlation analysis and ANOVAs were conducted to examine whether age, education, nationality, method of blood sugar control, and type of medication intake influence our core variables. Differences of education on NFC [*F* (7, 69) = 5.69, *p* < .0001], impulsivity [*F* (7, 69) = 9.28, *p* < .001], self-efficacy [*F* (7,69) = 3.16, *p* = .006] and self-management [*F* (7, 69) = 3.46, *p* = .003] were found. Thus, sex and education were controlled in the mediation analyses. Since years of illness (duration) correlated weakly with diabetes self-management (*r* = -.25, *p* = .03) it was also controlled in the mediation analysis.

### Relationship of impulsivity and NFC with diabetes self-management and HbA_1c_

Correlation analyses are shown in Table 2. Hypotheses 1 and 2 can be accepted because impulsivity strongly negatively correlated with diabetes self-management (*r* = −.58, *p* = <.001) and positively with HbA_1c_ (*r* = .40, *p* = <.001). Regarding the latter, lower HbA_1c_ values represents better glycemic control, and thus, positive correlations indicate worse glycemic control for individuals with higher impulsivity. In contrast, NFC strongly positively correlated with self-management (*r* = .57, *p* = <.001) and negatively with HbA_1c_ (*r* = −.52, *p* = <.001), that is, higher NFC was associated with better self-management and glycemic control.

**Table 2:** Intercorrelations of self-report instruments.

Variable	2	3	4	5
1 HbA_1c_	.40***	−.59***	−.51***	−.52***
2 BIS-11		−.46***	−.49***	−.58***
3 SDSCA			.75***	.57***
4 DMSES				.67***
5 NFC-K				−

*Annotations*. *HbA*_1c_ = *indicator for glycemic control (lower values indicate better glycemic control), BIS-11 = Barrat impulsiveness scale, SDSCA = Summary of Diabetes Self-care Activities Measure, DMSES = diabetes management self-efficacy scale, NFC-K = Need for Cognition short scale, Duration = Duration of Illness*.

* *p < .05*,

** *p < .01*,

*** *p < .001*.

HbA_1c_ was also negatively related to diabetes self-efficacy (*r* = −.51, *p* = <.001) and diabetes self-management (*r* = −.59, *p* < .001), indicating that individuals with better glycemic control reported higher diabetes self-efficacy and better self-management.

### Mediation analysis

**Fig 1:**
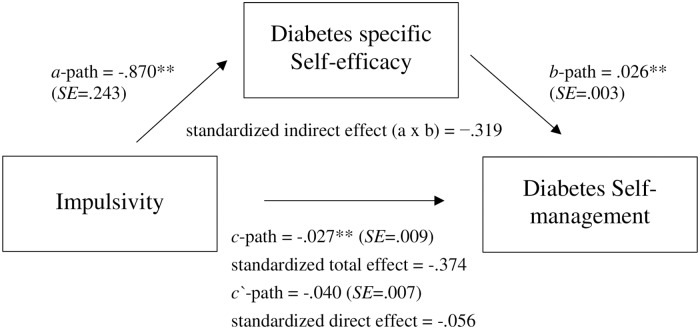
Relationship between impulsivity and diabetes self-management mediated by diabetes-specific self-efficacy. *p < .05, **p < .01.

To test hypothesis 3, two separate mediation models were conducted with impulsivity (Model 1) and NFC (Model 2), respectively, as independent variable. Since NFC and impulsivity are measured on different answering scales, the standardized regression coefficients of the direct and indirect effects are considered to be able to compare the effect sizes of both models. Model 1, as seen in Fig 1 (see also upper part of S1 Table in the supplementary material) showed a negative indirect effect of impulsivity on diabetes self-management through diabetes-specific self-efficacy. Higher impulsivity was related to lower self-efficacy, which in turn was related to decreased self-management [*β* = −.319, 95% BCa confidence intervals (*CI*) = −.487 to −.153]. The model gives evidence for full mediation as the direct effect of impulsivity on self-management [*β*_*c*_ = −.374] nearly vanished when the mediator was considered [*β*_*c’*_ = −.056].

**Fig 2:**
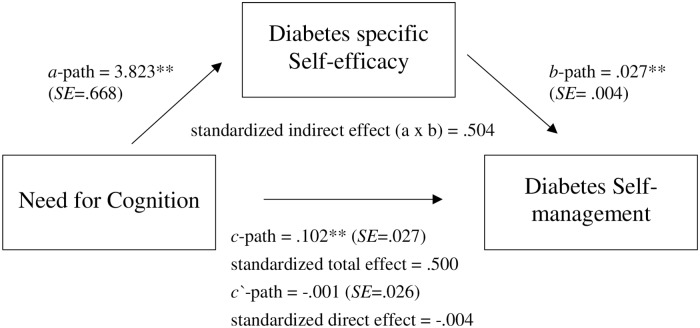
Relationship between Need for Cognition and diabetes self-management mediated by diabetes-specific self-efficacy. *p < .05, **p < .01.

Model 2 (see Fig 2 or lower part of S1 Table in the supplementary material) showed a positive indirect effect between NFC and diabetes self-management via diabetes-specific self-efficacy. Here, higher NFC was related to higher self-efficacy, which in turn was related to increased self-management [*β* = .504, 95% BCa *CI* = .325 to .695]. Similarly to Model 1, full mediation was apparent as the direct effect of NFC on self-management [*β*_*c*_ = .500] disappeared when the mediator was considered [*β*_*c’*_ = −.004].

**Fig 3:**
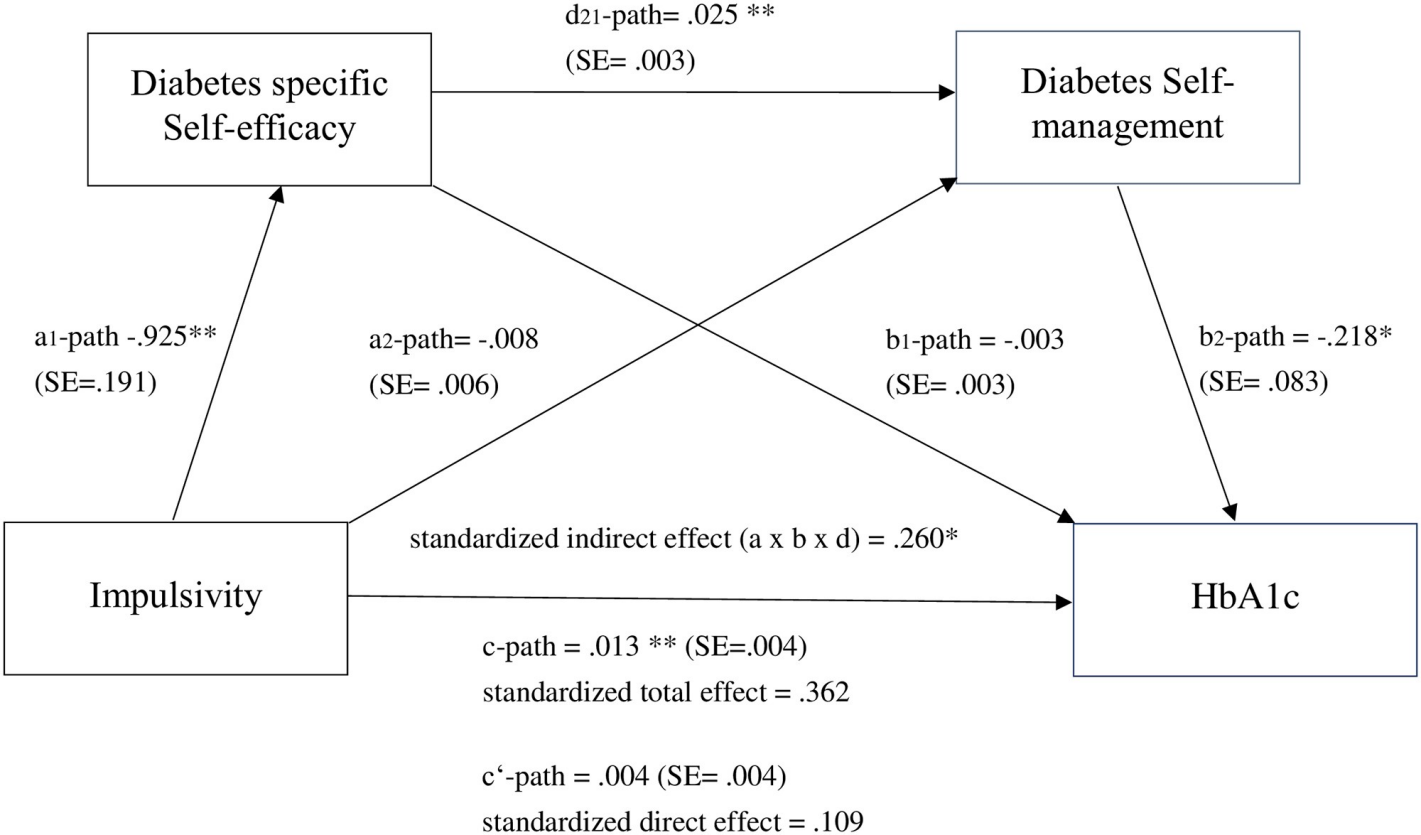
Relationship between impulsivity and HbA_1c_ mediated by self-efficacy and self-management. *p < .05, **p < .01.

**Fig 4:**
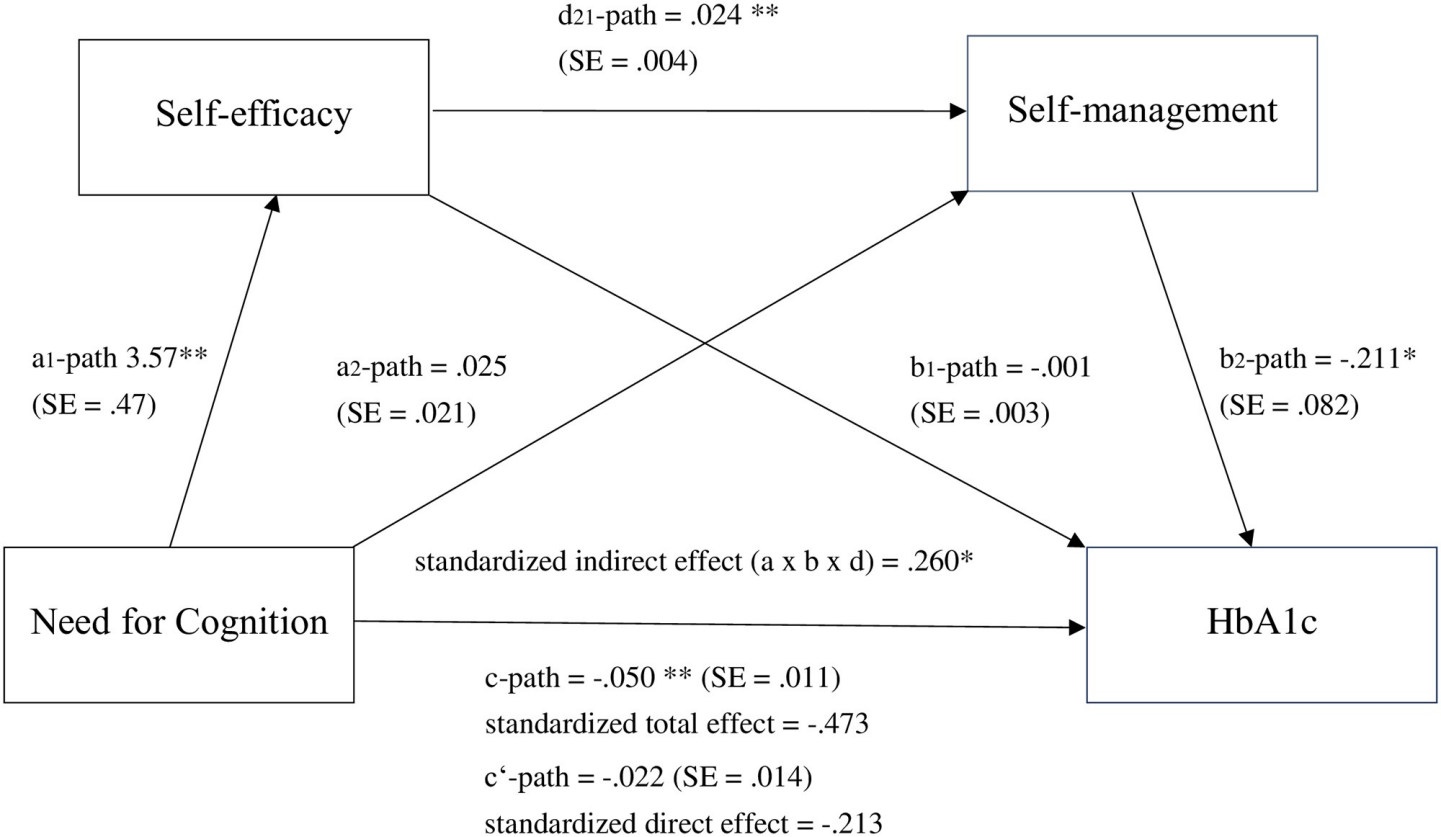
Relationship between Need for Cognition and HbA_1c_ mediated by self-efficacy and self-management. *p < .05, **p < .01.

In addition, it was examined whether impulsivity and NFC influence glycemic control (HbA_1c_) via the two mediators, diabetes-specific self-efficacy and diabetes self-management, whereby two serial mediation models were performed. It was assumed that impulsivity and NFC, respectively, influence self-efficacy, which in turn influences self-management, which in turn affects HbA_1c_. As given in Fig 3 for impulsivity and Fig 4 for NFC (as well as in S2 Table in the supplementary material), impulsivity [*β*_*c*_ = .362] and even with larger effect size NFC [*β*_*c*_ = -.473] significantly explained variance in HbA_1c_. As indicated by the direct effects c’ of serial mediation Model 1 [*β*_*c’*_ = .109] and serial mediation Model 2 (*β*_*c’*_ = -.213), the effects of impulsivity and NFC, respectively, on HbA_1c_ strongly decreased and became insignificant when the two mediators were added in the models providing evidence for partial mediation.

Furthermore, the variance of HbA_1c_ explained by the respective independent variable and the two mediators is similar for serial mediation Model 1 regarding impulsivity [*R*^*2*^ = .32, *F* (3,68) = 11.02, *p* = < .001] and serial mediation Model 2 regarding NFC [*R*^*2*^ = .34, *F* (3,68) = 11.84, *p* = < .001] as independent variable.

## Discussion

The goal of this paper was to examine the role of personality traits as potential protective and risk factors, respectively, on diabetes self-management and glycemic control as well as the mediational processes through which these personality traits might exert their influence. We found substantial links between NFC, impulsivity and diabetes-specific self-efficacy on diabetes self-management and glycemic control (HbA_1c_). As hypothesized, we found NFC to be strongly positively and impulsivity to be strongly negatively related to diabetes self-management. Moreover, we found higher NFC scores and lower impulsivity scores to be strongly associated with a better glycemic control as indicated by the HbA_1c_. All effects were of moderate to high effect sizes while the associations of NFC with diabetes self-management and HbA_1c_ were larger than that of impulsivity. Our hypothesis that these effects would be further mediated by diabetes-specific self-efficacy was also confirmed by mediation analysis.

In our model, higher NFC was associated with higher self-efficacy, which in turn was positively associated with self-management. In contrast, higher impulsivity was associated with lower self-efficacy, which in turn was correlated with poorer diabetes self-management. Thus, our findings may suggest that NFC acts as a protective factor and impulsivity as a potential risk factor influencing diabetes self-management, and consequently glycemic control in either a positive or negative way by increasing or decreasing diabetes-specific self-efficacy.

The facilitating effect of NFC might be explained by the general tendency of individuals high in NFC to be focused on task- or goal-relevant information and their higher cognitive motivation and task adherence. In earlier studies on a variety of chronic diseases, particularly type 2 diabetes, goal-orientation was identified as a predictor of improved adherence to medication, blood glucose monitoring and glycemic control as well as of self-efficacy [15, 64, 65]. Specifically, people high in NFC tend to be less likely to give up (i.e., are more persistent), are willing to make valid decisions on the basis of central or decision-relevant information and actively try new problem-solving approaches [42, 66], which could bolster their abilities to cope with the requirements and difficulties of managing their disease.

Impulsivity on the other hand is associated with reduced planning, foresight, and goal-orientation, which might lead to a reduction of self-management and glycemic control [32, 67]. Further, individuals with higher impulsivity show an inability to stay focused on a task especially in the presence of stressors or distractions [68]. This may drive individuals to repeatedly perform self-management tasks insufficiently, especially in challenging situations. Consequently, their perception to master such situations in the future (i.e., their self-efficacy) sinks which in turn might hinder their motivation to adhere to effective disease-management. In general, our results concur with other studies suggesting a detrimental role of impulsivity in health behavior and various diseases [22, 33, 55].

The fact that self-efficacy played a significant role in both simple mediation models and explained all (in the case of NFC) or nearly all (in the case of impulsivity) of the variance of the direct effect underscores the importance of self-efficacy in the treatment of type 2 diabetes. This is in line with several studies which come to similar conclusions regarding the important role of self-efficacy as a mediator for glycemic control and self-management behaviors in type 2 patients [47, 49, 50, 52, 53].

With respect to the serial mediation models with glycemic control as dependent variable, the mediating effect of self-efficacy was somewhat less pronounced. Both models showed remaining direct effects after the inclusion of the mediation variables leading to the assumption that further variables may mediate the effect of NFC and impulsivity, respectively, on HbA_1c_. A possible explanation for this pattern might be that glycemic control, can be influenced by several other factors such as psychophysiological factors like stress [69] or biological factors like insufficient fructose in the bloodstream [70]. Moreover, the negative relationship between diabetes self-management and HbA_1c_ as indicated by the outcome of the present study is in line with previous results identifying diabetes self-management as a predictor for glycemic control [19, 20, 31].

There are several strengths of our study. Studies on the relationship between impulsivity and diabetes-self management or glycemic control are rare and to our knowledge no study exists so far examining the relationships between NFC and these variables. The role of these personality traits in diabetes self-management and glycemic control may aid in expanding our understanding of personality-specific processes on successful diabetes treatment. For example patients with higher impulsiveness and lower NFC scores, respectively, could profit from an easily applicable questionnaire-based screening and a tailored treatment along their behavioral tendencies with the goal of increasing their self-efficacy. This, in turn, may reduce the negative effects of impulsivity and low NFC on their diabetes self-management and glycemic control. Because the screening for these traits is time efficient, medical practices could also assign patients of differing levels of NFC and impulsivity to group interventions. Such interventions are the most common form of training for diabetics, so that particularly patients high in NFC may support or advise patients with low NFC and/or high impulsivity, as in addition to the behavioral attributes outlined above, NFC is also associated with perceived competency and knowledge [42]. Additionally, it could be worthwhile to develop internet interventions following the example of Nikoloudakis, Crutzen [71], which tailored the way of presenting the information in accordance to the individuals’ level of need for cognition.

There are several limitations of our study. Because of a cross-sectional design, a causal interpretation of the results is not suggested. For example, in the context of impulsivity, hypoglycemia may influence acute impulsivity [38,39]. However, whether and to what extent such effects can lead to long-term changes in the basic personality factor impulsivity or what the variance proportion of such an influence on the overall personality variance proportion could be, is not yet clear and requires appropriate prospective analyses in the future. Moreover, there is also comparatively good evidence from previous studies that impulsivity or related personality traits are potential risk factors for the development of type 2 diabetes or behaviors which are risk factors for later developing diabetes [29, 34–36]

Moreover, self-reports may be influenced by social desirability and may thus be complemented by observer ratings in future research. However, since the questionnaires were answered online and patients were anonymous social desirability is assumed to play a minor role [72]. Finally, generalization of our results is limited to German type 2 diabetes patients.

## Conclusion

Our study suggests that the personality traits NFC and impulsivity are associated with diabetes self-management and glycemic control. The findings indicate that NFC increases self-management and glycemic control, while the opposite is true for impulsivity. These effects were significantly mediated by diabetes-specific self-efficacy. Since the examined traits could act either as protective or risk factors for type 2 diabetes patients, an easy-to-use questionnaire-based patient screening might be conducted to foster diabetes treatment through the development of trait-tailored interventions. This, in turn, may improve diabetes self-management and glycemic control.

## Supporting information

S1 Table Regression coefficients of the models regarding the effect of impulsivity (Model 1) and NFC (Model 2) on Diabetes self-management mediated by diabetes specific self-efficacy. *Annotations*. *N* = 77. R^2^Y,X represents the proportion of variance in Y explained by X; R^2^M,X represents the proportion of variance in M explained by X, and R^2^Y,MX represents the proportion of variance in Y explained by X and M. The 95% confidence intervals (CI) for the indirect effect were calculated with the bias-corrected bootstrapping method including 5000 resamples. BIS as measure of impulsivity represents the predictor variable (X) in Model 1, NFC as measure of Need for Cognition represent the predictor variable (X) for Model 2, SDSCA as measure of diabetes self-management represents the dependent variable (Y) in both models und DMSES as measure of diabetes specific self-efficacy represents the mediator variable (M) in both models.
(DOCX)

S2 Table Regression results for the serial mediation of the effects of NFC and impulsivity on glycemic control mediated by diabetes specific self-efficacy and diabetes self-management. *Annotations N* = 72. R^2^Y,X represents the proportion of variance in Y explained by X, R^2^M1,X represents the proportion of variance in M1 which is explained by X, R^2^M2,M1X represents the proportion of variance in M2 which is explained by X and M1, R^2^Y,M1m2X represents the proportion of variance in Y which is explained by X, M1 and M2. The 95% confidence intervals (CI) for the indirect effect were calculated with the bias-corrected bootstrapping method including 5000 resamples. BIS as measure of impulsivity represents the predictor variable (X) in Model 1, NFC as measure of Need for Cognition represent the predictor variable (X) for Model 2, HbA_1c_ as a measure of glycemic control represents the dependent variable (Y) in both models. DMSES as measure of diabetes specific self-efficacy represents the mediating variable (M1) in both models and SDSCA as measure of diabetes self-management represents the second mediating variable (M2) for both models.
(DOCX)
