# Supplementary material for: Individual differences in impulsivity and need for cognition as potential risk or resilience factors of diabetes self-management and glycemic control
Source: PLoS One. 2020 Jan 29;15(1):e0227995. doi: 10.1371/journal.pone.0227995 (PMC6988919; doi:10.1371/journal.pone.0227995)
Supplement: S1 Table — Annotations. N = 77. R2Y,X represents the proportion of variance in Y explained by X; R2M,X represents the proportion of variance in M explained by X, and R2Y,MX represents the proportion of variance in Y explained by X and M. The 95% confidence intervals (CI) for the indirect effect were calculated with the bias-corrected bootstrapping method including 5000 resamples. BIS as measure of impulsivity represents the predictor variable (X) in Model 1, NFC as measure of Need for Cognition represent the predictor variable (X) for Model 2, SDSCA as measure of diabetes self-management represents the dependent variable (Y) in both models und DMSES as measure of diabetes specific self-efficacy represents the mediator variable (M) in both models. (DOCX) [file pone.0227995.s001.docx]

**Supporting Material**

S1 Table. Regression coefficients of the models regarding the effect of impulsivity (Model 1) and NFC (Model 2) on Diabetes self-management mediated by diabetes specific self-efficacy.

| Model | Estimate | *SE* | *p* | *CI*  (lower limit) | *CI*  (upper limit) |
| --- | --- | --- | --- | --- | --- |
| Model 1 without Mediator |  |  |  |  |  |
| BIS 🡪 SDSCA (c) | −.0268 | .0092 | .0047 | −0.0450 | −0.0085 |
| R²Y,X | .3034 (p = <.001) | | | |  |
| Model 1 with Mediator |  |  |  |  |  |
| BIS 🡪 DMSES (a) | −.8701 | .2433 | .0006 | −1.1355 | −0.3849 |
| DMSES 🡪 SDSCA (b) | .0262 | .0032 | <.0001 | 0.0198 | 0.0327 |
| BIS 🡪 SDSCA (c´) | −.0040 | .0072 | .5826 | −0.0183 | 0.0104 |
| Indirect Effect (a x b) | −.0281 |  |  | −0.0350 | −0.0111 |
| Standardized indirect effect | −.3187 | .0845 |  | −0.4872 | −0.1530 |
| R²M,X | .3507 (p = <.001) | | | |  |
| R²Y,MX | .6410 (p = <.001) | | | |  |
| Model 2 without Mediator |  | | | |  |
| NFCK 🡪 SDSCA (c) | .1023 | .0270 | .0003 | 0.0484 | 0.1562 |
| R²Y,X | .3507 (p = <.001) | | | |  |
| Model 2 with Mediator |  |  |  |  |  |
| NFC 🡪 DMSES (a) | 3.8226 | .6680 | <.0001 | 2.4906 | 5.1546 |
| DMSES 🡪 SDSCA (b) | .0270 | .0036 | <.0001 | 0.0198 | 0.0342 |
| NFC 🡪 SDSCA (c´) | −.0008 | .0245 | .9738 | −0.0497 | 0.0481 |
| Indirect Effect (a x b) | .1034 |  |  | 0.0636 | 0.1459 |
| Standardized indirect effect | .5038 | .0953 |  | 0.3246 | 0.6950 |
| R²M,X | .4756 (p = <.001) | | | |  |
| R²Y,MX | .6394 (p = <.001) | | | |  |

*Annotations. N =* 77. R²Y,X represents the proportion of variance in Y explained by X; R²M,X represents the proportion of variance in M explained by X, and R²Y,MX represents the proportion of variance in Y explained by X and M. The 95% confidence intervals (CI) for the indirect effect were calculated with the bias-corrected bootstrapping method including 5000 resamples. BIS as measure of impulsivity represents the predictor variable (X) in Model 1, NFC as measure of Need for Cognition represent the predictor variable (X) for Model 2, SDSCA as measure of diabetes self-management represents the dependent variable (Y) in both models und DMSES as measure of diabetes specific self-efficacy represents the mediator variable (M) in both models.
