# Supplementary material for: Individual differences in impulsivity and need for cognition as potential risk or resilience factors of diabetes self-management and glycemic control
Source: PLoS One. 2020 Jan 29;15(1):e0227995. doi: 10.1371/journal.pone.0227995 (PMC6988919; doi:10.1371/journal.pone.0227995)
Supplement: S2 Table — Annotations N = 72. R2Y,X represents the proportion of variance in Y explained by X, R2M1,X represents the proportion of variance in M1 which is explained by X, R2M2,M1X represents the proportion of variance in M2 which is explained by X and M1, R2Y,M1m2X represents the proportion of variance in Y which is explained by X, M1 and M2. The 95% confidence intervals (CI) for the indirect effect were calculated with the bias-corrected bootstrapping method including 5000 resamples. BIS as measure of impulsivity represents the predictor variable (X) in Model 1, NFC as measure of Need for Cognition represent the predictor variable (X) for Model 2, HbA1c as a measure of glycemic control represents the dependent variable (Y) in both models. DMSES as measure of diabetes specific self-efficacy represents the mediating variable (M1) in both models and SDSCA as measure of diabetes self-management represents the second mediating variable (M2) for both models. (DOCX) [file pone.0227995.s002.docx]

S2 Table. Regression results for the serial mediation of the effects of NFC and impulsivity on glycemic control mediated by diabetes specific self-efficacy and diabetes self-management.

| Model | Estimate | SE | p | CI  (lower limit) | CI  (upper limit) |
| --- | --- | --- | --- | --- | --- |
| Model 1 without Mediator |  |  |  |  |  |
| BIS 🡪 HbA_1c_ (c) | .0131 | .0040 | .0018 | 0.0051 | 0.0212 |
| R²Y,X | .1310 (p = .001) | | | |  |
| Model 1 with mediators |  |  |  |  |  |
| BIS 🡪 DMSES (a1) | −.9250 | .1908 | <.0001 | −1.3055 | −0.5444 |
| DMSES 🡪 SDSCA (d21) | .0252 | .0033 | <.0001 | 0.0187 | 0.0318 |
| BIS 🡪 SDSCA (a2) | −.0077 | .0061 | .2097 | −0.0198 | 0.0044 |
| DMSES 🡪 HbA_1c_ (b1) | −.0026 | .0031 | .4016 | −0.0087 | 0.0035 |
| SDSCA 🡪 HbA_1c_ (b2) | −.2180 | .0826 | .0103 | −0.3829 | −0.0530 |
| BIS 🡪 HbA_1c_ (c´) | .0039 | .0042 | .3520 | −0.0045 | 0.0124 |
| Total indirect effect | .0092 | .0023 |  | 0.0044 | 0.0137 |
| Standardized total indirect effect | .2529 | .0682 |  | 0.1172 | 0.0207 |
|  |  |  |  |  |  |
| R²M1,X | .2513 (p = <.001) | | | |  |
| R²M2,M1X | .5763 (p = <.001) | | | |  |
| R²Y,M1M2X | .3217 (p = <.001) | | | |  |
| Model 2 without Mediator |  | | | |  |
| NFCK 🡪 HbA_1c_ (c) | −.0498 | .0111 | <.0001 | −0.0719 | −0.0277 |
| R²Y,X | .2234 (p = <.001) | | | |  |
| Model 2 with mediators |  |  |  |  |  |
| NFC 🡪 DMSES (a1) | 3.5700 | .4786 | <.0001 | 2.6154 | 4.5247 |
| DMSES 🡪 SDSCA (d21) | .0242 | .0038 | <.0001 | 0.0166 | 0.0318 |
| NFC 🡪 SDSCA (a2) | .0250 | .0205 | .2253 | −0.0158 | 0.0659 |
| DMSES 🡪 HbA_1c_ (b1) | −.0011 | .0033 | .7395 | −0.0076 | 0.0054 |
| SDSCA 🡪 HbA_1c_ (b2) | −.2106 | .0816 | .0120 | −0.3734 | −0.0478 |
| NFC 🡪 HbA_1c_ (c´) | −.0224 | .0140 | .1134 | −0.0504 | 0.0055 |
| Total indirect effect | −.0274 | .0111 |  | −0.0508 | −0.0077 |
| Standardized total indirect effect | −.2598 | .1042 |  | −0.4795 | −0.0701 |
| R²M1,X | .4428 (p = <.001) | | | |  |
| R²M2,M1X | .5757 (p = <.001) | | | |  |
| R²Y,M1M2X | .3431 (p = <.001) | | | |  |

*Annotations N =* 72. R²Y,X represents the proportion of variance in Y explained by X , R²M1,X represents the proportion of variance in M1 which is explained by X, R²M2,M1X represents the proportion of variance in M2 which is explained by X and M1, R²Y,M1m2X represents the proportion of variance in Y which is explained by X, M1 and M2. The 95% confidence intervals (CI) for the indirect effect were calculated with the bias-corrected bootstrapping method including 5000 resamples. BIS as measure of impulsivity represents the predictor variable (X) in Model 1, NFC as measure of Need for Cognition represent the predictor variable (X) for Model 2, HbA_1c_ as a measure of glycemic control represents the dependent variable (Y) in both models. DMSES as measure of diabetes specific self-efficacy represents the mediating variable (M1) in both models and SDSCA as measure of diabetes self-management represents the second mediating variable (M2) for both models.
